# Case report: a rare case of left-sided sinus of Valsalva aneurysm with concomitant coronary artery fistula and carotid artery aneurysm

**DOI:** 10.1093/ehjcr/ytaf508

**Published:** 2025-10-03

**Authors:** Marsioleda Kemberi, Gentjan Jakaj, Richard Stratton, Daryll Baker, Olaf Wendler

**Affiliations:** Barts and the London Medical School, Queen Mary University of London, Mile End Road, London E1 4NS, UK; Department of Cardiothoracic Surgery, King's College Hospital, Golden Jubilee, London SE5 9RS, UK; Heart, Vascular and Thoracic Institute, Cleveland Clinic, 33 Grosvenor Place, London SW1X 7HY, UK; General Practice Institute, Cleveland Clinic, 33 Grosvenor Place, London SW1X 7HY, UK; Heart, Vascular and Thoracic Institute, Cleveland Clinic, 33 Grosvenor Place, London SW1X 7HY, UK; Heart, Vascular and Thoracic Institute, Cleveland Clinic, 33 Grosvenor Place, London SW1X 7HY, UK

**Keywords:** Case report, Marfan’s, Left sinus of Valsalva, Coronary fistula

## Abstract

**Background:**

Sinus of Valsalva aneurysms (SVAs) are rare cardiac anomalies often occurring with co-existing cardiac lesions, such as ventricular septal defects and aortic regurgitation. While often asymptomatic, SVAs can lead to severe complications, including rupture, aortic regurgitation, and dissection. Carotid aneurysms are also uncommon and pose a significant stroke risk if left untreated.

**Case summary:**

We report the case of a 56-year-old woman who initially presented with a pulsatile right neck mass and was diagnosed with a right internal carotid artery aneurysm. Subsequent imaging revealed a 45 mm left-sided sinus of Valsalva aneurysm (SVA) and a concomitant coronary artery fistula involving the right coronary artery. She underwent staged surgical management with a modified Bentall procedure and later excision of the carotid aneurysm. Post-operative recovery was uneventful. Genetic testing later confirmed a diagnosis of Marfan's syndrome.

**Discussion:**

SVAs originating from the left coronary sinus are exceedingly rare, but their potential for fatal complications demands early intervention. This case demonstrates that surgical repair of the left SVA, associated coronary fistula, and carotid aneurysm is both safe and effective, and it reinforces the role of genetic testing in patients with atypical presentations of aneurysmal disease. Multimodal imaging and a multidisciplinary approach are essential in guiding treatment decisions in such unusual presentations.

Learning points

- A thorough vascular evaluation and multidisciplinary approach, including multimodal imaging and genetic testing, is essential in patients with suspected connective tissue disorders, even when classical phenotypic features are absent.
- Early surgical intervention provides excellent outcomes in patients with left sinus of Valsalva aneurysm with concomitant coronary artery fistula and carotid artery aneurysm which are rare but clinically significant vascular anomalies.

## Introduction

Right-sided sinus of Valsalva aneurysms (SVAs) are most common and often present with obstruction of the right ventricular outflow tract or even a rupture into the right ventricle. SVAs with an origin from the left coronary sinus aneurysms are exceedingly rare and usually present with no or unspecific clinical symptoms. Surgical treatment is the standard of care to prevent SVA rupture, dissection, or aortic regurgitation which can be life-threatening.

## Summary figure

**Figure:**
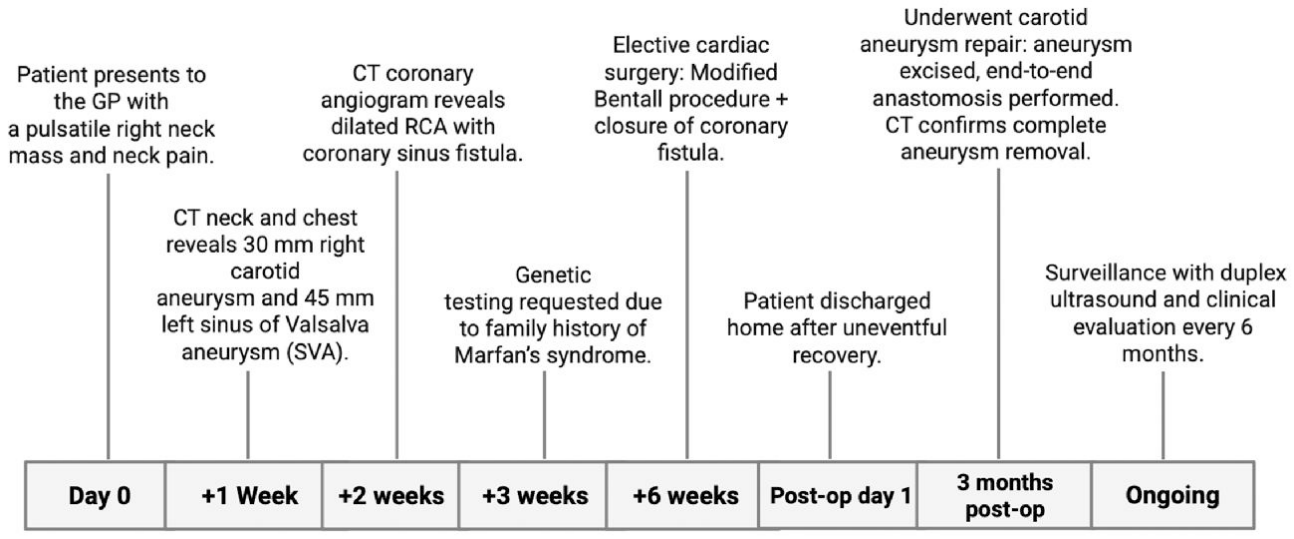


## Case presentation

This 56-year-old female presented initially to her general practitioner and vascular surgeon with a pulsatile mass on her right neck, which caused some neck pain. The patient has a family history of Marfan's syndrome, with both her father and brother affected. However, she did not exhibit a typical Marfanoid habitus. There was no evidence of pectus deformity, myopia, or high arched palate. Nevertheless, the presence of long, slender fingers and joint hypermobility in the hands and knees raised suspicion of an underlying connective tissue disorder.

**Figure 1:**
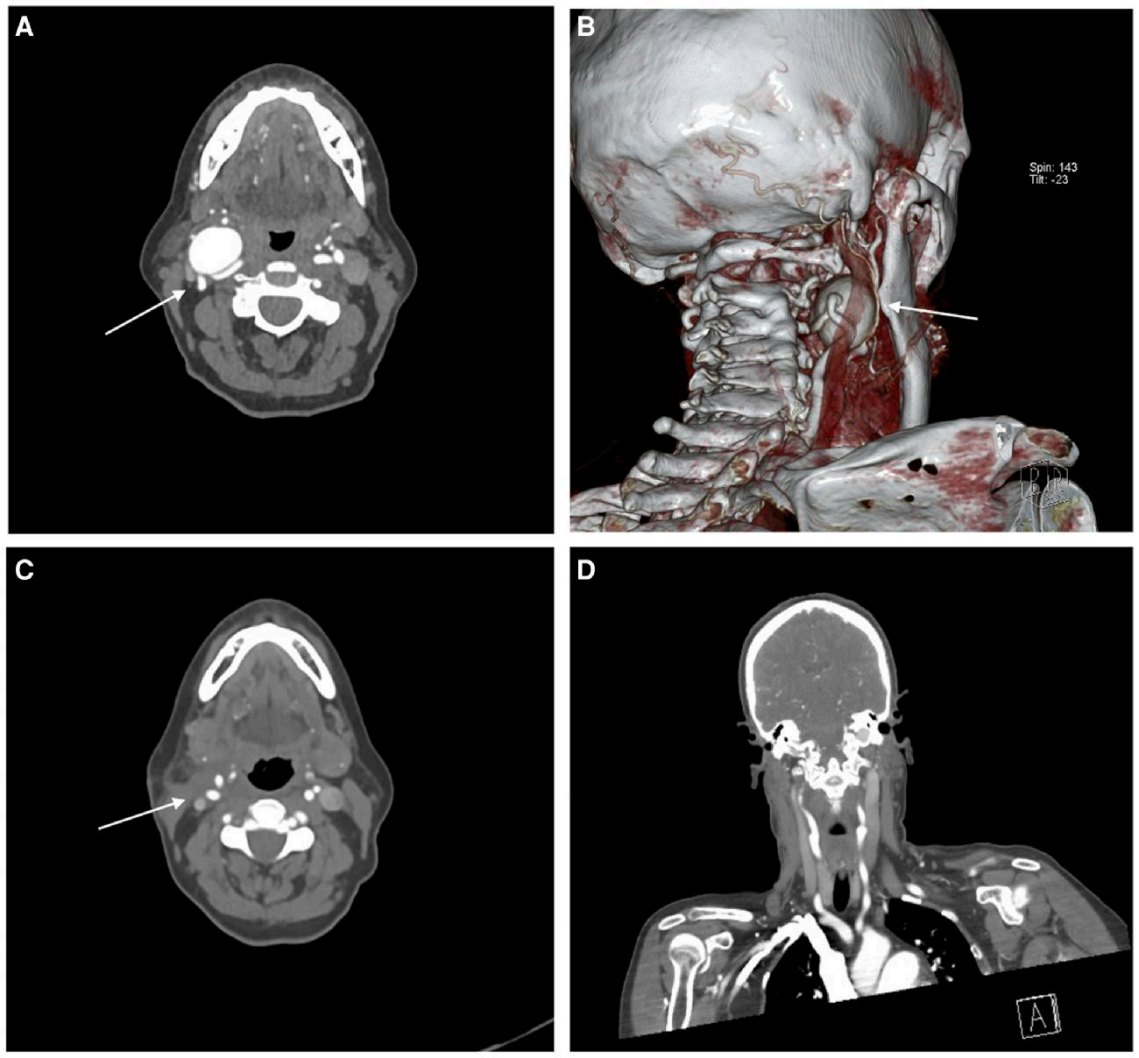
Representative computed tomography images and a three-dimensional reconstruction of the neck and upper cervical region in a patient with an internal carotid aneurysm. (*A*) Axial computed tomography slice demonstrating the oropharyngeal area and adjacent vascular structures, including the right internal carotid aneurysm (arrow). (*B*) 3D volume-rendered reconstruction clearly outlining the internal carotid aneurysm (arrow) and the rest of the arterial anatomy of the neck and skull base. (*C*) Axial computed tomography image obtained after aneurysm repair, showing successful restoration of the arterial lumen and improved vessel contour. (*D*) Coronal view providing a front-to-back overview of the upper cervical region, including the course of the carotid vessels, post-aneurysmal repair.

**Figure 2:**
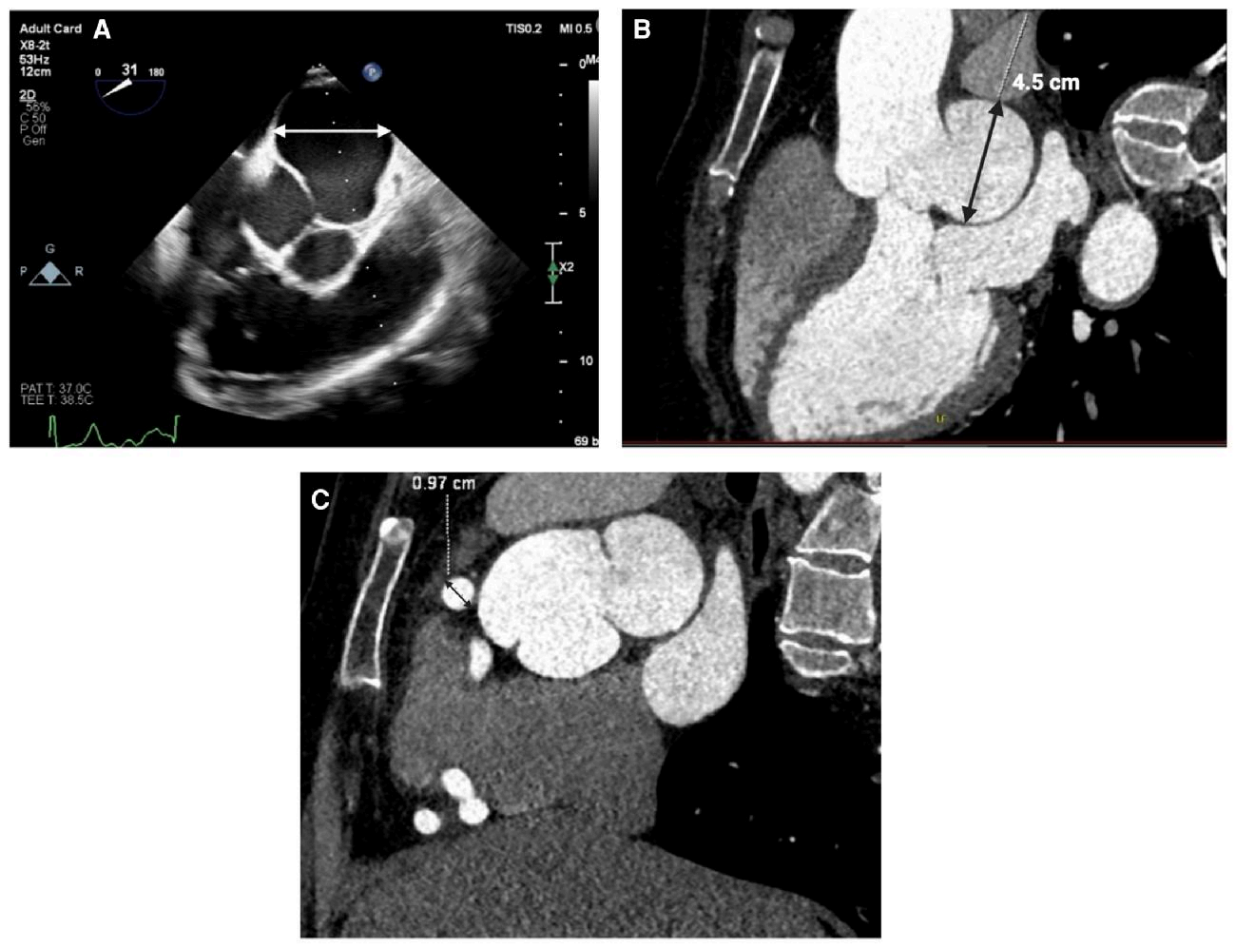
Multimodal imaging of a left Sinus of Valsalva aneurysm. (*A*) Transthoracic echocardiogram illustrating the aortic valve and aortic root in real time, allowing functional and haemodynamic assessment, and clearly showing the dilated left sinus of Valsalva. (*B*) Contrast-enhanced computed tomography scan measuring the left sinus of Valsalva at 45 mm, confirming sinus dilatation. (*C*) Computed tomography coronary angiogram showing dilated right coronary artery at 9.7 mm, due to a coronary fistula communicating with the coronary sinus.

Initial differential diagnoses included carotid artery aneurysm, arteriovenous malformation, and cervical lymphadenopathy. Her neck and chest computed tomography (CT) scan revealed a 30 mm carotid artery aneurysm (*Figure 1*), and in addition, a 45 mm left SVA was found (*Figure 2*). Diameters of the mid-ascending aorta measured 35 mm with normal diameters in the aortic arch, descending, and abdominal aorta.

Her transthoracic echocardiography (TTE) confirmed a left SVA of 45 mm and a total mid-sinus aortic root diameter of 62 mm. The aortic valve was tricuspid, with mild central aortic regurgitation, and left ventricular function was normal (ejection fraction of 59%). Her CT coronary angiogram (*Figure 2*) showed no evidence of obstructive coronary artery disease, but a tortuous and dilated right coronary artery (RCA) with a coronary fistula communicating with the coronary sinus. Genetic testing for connective tissue disorders was requested at the same time.

**Figure 3:**
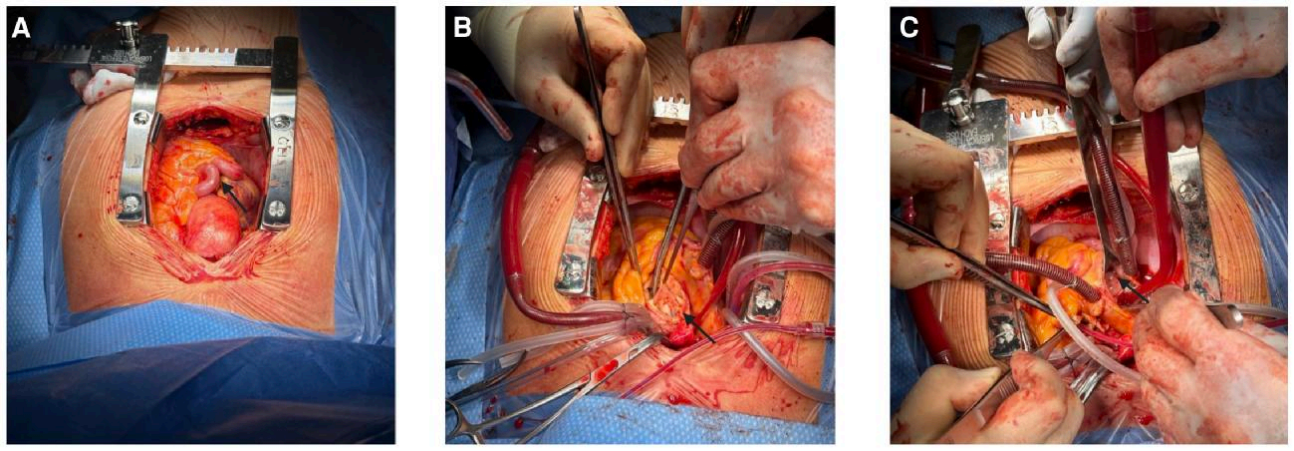
Intraoperative views illustrating the surgical repair of a left sinus of Valsalva aneurysm. (*A*) Tortuous and dilated right coronary artery (arrow) and dilated ascending aorta upon opening the pericardium. (*B*) Arrow pointing to the dilated left coronary sinus of Valsalva. (*C*) Fistula repair, identification by giving cardioplegia to the left main stem ostium.

Elective surgery was planned for aortic root ± aortic valve replacement for prognostic reasons. After a median sternotomy, the pericardium was opened, revealing a mildly dilated ascending aorta. The RCA was severely dilated and tortuous (*Figure 3*). Cardiopulmonary bypass was instituted (right atrium to proximal aortic arch), and the patient was cooled down to 32°. After the cross clamp was applied, the ascending aorta was opened through a longitudinal incision, and a native tricuspid aortic valve with large fenestrations in the left coronary cusp (LCC) was found. Therefore, given the age of the patient, the decision was made to replace the aortic valve. A modified Bentall procedure was performed using a 27 mm Inspiris™ bioprosthetic valve (Edwards Lifesciences, Irvine, CA, USA) and a 30 mm Gelweave Valsalva conduit (VASCUTEK TERUMO, Renfrewshire, Scotland, UK). The right atrium was opened, and the coronary fistula was identified by giving cardioplegia and direct observation of the coronary sinus ostium, where the exit of the fistula was found. This was closed using a 7-0 Prolene suture. The patient was successfully weaned off bypass with no need for inotropic support. The intraoperative TEE showed low mean/peak gradients, no regurgitation, and a normal left ventricular function.

Post-operatively, the patient was stable with minimal noradrenaline requirements. She was extubated without issues. Initially, she showed atrio-ventricular conduction disturbances, which needed intermittent pacing. Finally, she went back into a stable sinus rhythm and enjoyed an uneventful recovery before she went home, 11 days after surgery. Her genetic testing confirmed a diagnosis of Marfan's syndrome and showed that she is suffering from an autosomal dominant inheritance of a FBN1-related condition.

Three months after recovering from her aortic root replacement, the patient underwent excision of her 30 mm right internal carotid artery aneurysm. Dissection preserved cranial nerves X, XI, and XII as well as the internal jugular vein. The aneurysm was resected and reconstructed via an end-to-end 6-0 Prolene anastomosis. She was initially on aspirin and apixaban, but aspirin was discontinued to reduce bleeding risk. A follow-up CT (*Figure 1*) confirmed complete aneurysm removal with no anastomotic stenosis, and she remains neurologically intact. Ongoing surveillance includes duplex ultrasounds and clinical evaluations every six months.

## Discussion

SVAs are rare anomalies, with an incidence of approximately 0.09% in the general population.^1^ They present significant clinical challenges due to the potential for rupture and association with co-existing cardiac lesions, such as ventricular septal defects (31%) and aortic regurgitation (44%).^1,2^ The most common origin of these aneurysms is the right coronary sinus (94%), followed by the non-coronary sinus (5%) and the left coronary sinus (1%).^2^ However, more recent literature suggests that left-sided involvement may account for up to 28% (15/53) of reported cases.^3^ Notably, only eight of those were isolated cases of left sinus involvement.^3^ This discrepancy likely reflects both historical underreporting and advances in diagnostic imaging techniques that have improved the identification of asymptomatic aneurysms. Although SVAs are often associated with Marfan's syndrome, isolated left coronary SVAs remain extremely rare with only a few reported cases.^4^ Extracranial carotid artery aneurysms (CAAs) are equally notable in their rarity and account for <4% of peripheral aneurysms and <1% of carotid disease.^5^

Most unruptured SVAs are asymptomatic and are often discovered incidentally during imaging studies performed for other reasons.^2^ If untreated, right-sided SVAs can lead to right ventricular outflow tract obstruction, arrhythmias, myocardial ischaemia due to compression of coronary arteries, but also aortic regurgitation, dissection, and perforation.^1,2^

The management of SVAs, particularly those that are unruptured, typically depends on their size, location, and associated symptoms. Surgical repair is the only treatment option, with a low perioperative risk and excellent long-term survival, reported at 95% over 10 years.^1,6^ Although specific guidelines for repair of SVAs are yet to be established, it is generally accepted to follow repair guidelines for aortic root aneurysms. The 2024 ESC guidelines emphasize that surgery is recommended for patients with dilatation of the aortic root or ascending aorta with a tricuspid aortic valve when the maximum diameter reaches ≥55 mm (Class I, Level A).^7^ However, in genetically confirmed Marfan’s syndrome, surgery is advised when the diameter reaches ≥45 mm, in the presence of high-risk features such as a family history of dissection. In most patients, the native aortic valve can be preserved by valve-sparing root or only sinus replacement. While sinus-only repair is our preferred surgical approach in patients with normal aortic valve anatomy and no connective tissue disease, the presence of large fenestrations in the LCC and the diagnosis of Marfan's disease supported the decision for a modified Bentall procedure in this case. In younger patients with a diagnosis of connective tissue disease in this context and no other cardiac comorbidities, we would have considered to replace the entire aortic root and preserve the native aortic valve with a David's procedure, to minimize future risk of aneurysmal degeneration and re-intervention. The coronary fistula was easily identified and therefore closed at the same time. The perioperative course was unremarkable, and the patient attended her 3 monthly clinic follow-up without any symptoms.

Carotid aneurysms may also occur in Marfan’s syndrome; however, similar to SVAs, there is a general lack of case–control and prospective studies investigating the true prevalence of these findings in these patient populations.^8^ Despite the high prevalence of atherosclerotic disease in extracranial carotid arteries, true CAAs are relatively rare, with an incidence reported at around 1% of all carotid surgeries.^5^ Aneurysmal change of the internal carotid artery in patients with connective tissue disorders has been reported but is commonly due to false aneurysm formation.^8^ The natural history of CAAs is generally poor if left untreated, mainly due to a significantly elevated stroke risk, despite rupture being comparatively uncommon.^5,9^ In this patient, excision of the 30 mm right internal carotid aneurysm was performed shortly after aortic root replacement, resulting in an uneventful recovery and no neurological deficits.

This case demonstrates that surgery provides excellent treatment in these complex patients and that comprehensive vascular evaluation and genetic testing should be standard in patients with aortic aneurysms, even if they don't show typical features of connective tissue disease. Importantly, this case highlights the value of genetic confirmation in patients with atypical phenotypes, as the diagnosis of Marfan’s syndrome significantly influenced both the timing and strategy of surgical intervention.

## Lead author biography


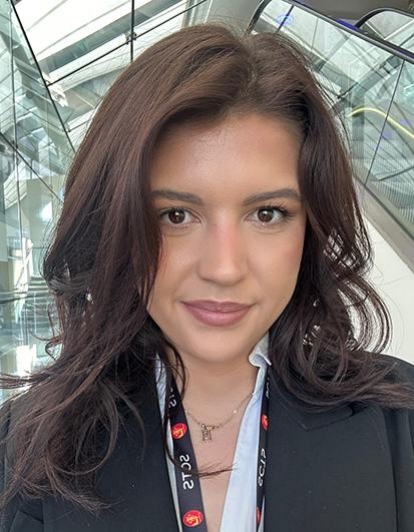


Marsioleda Kemberi is a resident doctor who graduated from Barts and The London School of Medicine and Dentistry with a strong interest in cardiothoracic surgery and aortic pathology. She hopes to pursue a career combining clinical practice with academic research in cardiovascular surgery.

## Data Availability

The data underlying this article are available in the article and in its online supplementary material.

## References

[1] Weinreich M, Yu P-J, Trost B. Sinus of Valsalva aneurysms: review of the literature and an update on management. Clin Cardiol 2015;38:185–189. 25757442 10.1002/clc.22359 PMC6711005

[2] Guo DW, Cheng TO, Lin ML, Gu ZQ. Aneurysm of the sinus of Valsalva: a roentgenologic study of 105 Chinese patients. Am Heart J 1987;114:1169–1177. 3673883 10.1016/0002-8703(87)90193-1

[3] Nguyen Q, Vervoort D, Phan K, Luc JGY. Surgical management for unruptured sinus of Valsalva aneurysms: a narrative review of the literature. J Thorac Dis 2021;13:1833–1850. 33841972 10.21037/jtd-20-2682 PMC8024852

[4] Chamsi-Pasha MA, Lawrie GM. Aneurysmal left sinus of Valsalva in Marfan’s syndrome. Eur Heart J 2018;39:285. 29020253 10.1093/eurheartj/ehx452

[5] Bush R, Long P, Atkins M. Carotid artery aneurysms. In: Rutherford’s Vascular Surgery and Endovascular Therapy. 9th ed. Amsterdam, Netherlands: Elsevier; 2018. p1248–1251.

[6] Yan F, Huo Q, Qiao J, Murat V, Ma S-F. Surgery for sinus of Valsalva aneurysm: 27-year experience with 100 patients. Asian Cardiovasc Thorac Ann 2008;16:361–365. 18812342 10.1177/021849230801600504

[7] Mazzolai L, Teixido-Tura G, Lanzi S, Boc V, Bossone E, Brodmann M, et al 2024 ESC guidelines for the management of peripheral arterial and aortic diseases. Eur Heart J 2024;45:3538–3700. 39210722 10.1093/eurheartj/ehae179

[8] Kim ST, Brinjikji W, Lanzino G, Kallmes DF. Neurovascular manifestations of connective-tissue diseases: a review. Interv Neuroradiol 2016;22:624–637. 27511817 10.1177/1591019916659262 PMC5564353

[9] Garg K, Rockman CB, Lee V, Maldonado TS, Jacobowitz GR, Adelman MA, et al Presentation and management of carotid artery aneurysms and pseudoaneurysms. J Vasc Surg 2012;55:1618–1622. 22341576 10.1016/j.jvs.2011.12.054

